# Contributions of the Bone Microenvironment to Monoclonal Gammopathy of Undetermined Significance Pathogenesis

**DOI:** 10.1007/s11914-018-0479-z

**Published:** 2018-09-18

**Authors:** Beatriz Gámez, Claire M. Edwards

**Affiliations:** 1 Nuffield Department of Surgical Sciences, University of Oxford, Oxford, UK; 2 Botnar Research Centre, Oxford, OX3 7LD UK; 3 Nuffield Department of Orthopaedics, Rheumatology and Musculoskeletal Sciences, University of Oxford, Oxford, UK

**Keywords:** MGUS, Myeloma, Bone, Fracture, Osteoblast, Osteoclast

## Abstract

**Purpose of Review:**

MGUS (monoclonal gammopathy of undetermined significance) is a plasma cell disorder characterized by a moderate increase in serum monoclonal protein (≤ 3 g/dL), an increase in bone marrow plasma cell infiltration (≤ 10%) and the absence of any end-organ damage. Although MGUS is considered a benign condition, evidence for clinical consequences is increasing. In this review, we examine the most recent evidence regarding MGUS manifestations and risks and present an overview of MGUS population studies as related to bone disease. Data reveals important MGUS-related bone alterations that may contribute to disease pathogenesis.

**Recent Findings:**

MGUS patients present a rate of 1% per year risk of progression to the more aggressive multiple myeloma (MM) and therefore research has focused on the study of risk factors and the events leading to this progression. However, the exact health implications of MGUS itself and the mechanisms behind them remain unclear.

**Summary:**

It is now evident that the bone microenvironment plays a key role in hematologic cancers and other oncogenic processes leading to bone metastasis.

## Introduction

Monoclonal gammopathy of undetermined significance (MGUS) is a premalignant condition characterized by the unexpected finding of monoclonal protein or M-protein in serum (≤ 3 g/dL) and moderate plasma cell proliferation (< 10%) in the bone marrow. No other signs of disease should be present in individuals diagnosed with MGUS although they will require lifelong follow-up. MGUS prevalence is approximately 3% of the population aged over 50 years and increases with age. Furthermore, it is thought that MGUS remains underdiagnosed due to the lack of evident signs [1, 2]. Although it was historically considered a benign condition, MGUS patients have a 1% per year risk of progression to multiple myeloma (MM) or other malignancies [3–5].

**Fig. 1:**
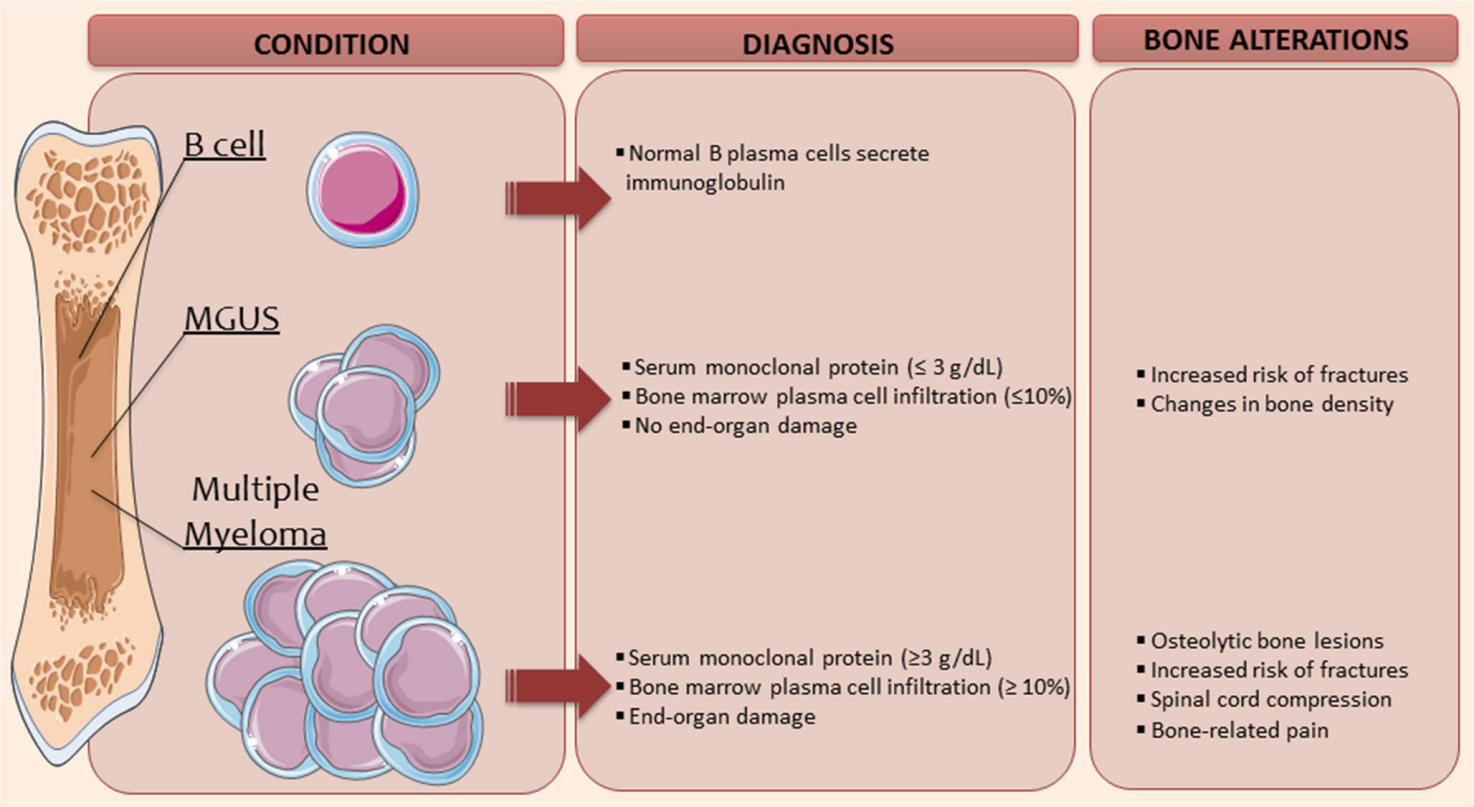
Bone changes in MGUS and myeloma. The diagnostic criteria of MGUS and myeloma are illustrated alongside the reported changes within the bone microenvironment

MM is a B cell neoplastic disease characterized by the presence of life-threating symptoms including renal failure, immunodeficiency, and osteolytic bone lesions (Fig. 1). All these secondary clinical manifestations arise from a more aggressive accumulation of malignant plasma cells throughout the bone marrow and higher levels of paraprotein in the serum as compared to MGUS patients. Four early oncogenic events have been described for MGUS and MM. These genetic changes include translocations, dysregulation of cyclin D/retinoblastoma pathway, hyperdiploidy, and chromosome 13 deletions [6]. The fact that MGUS patients carry most of the genomic complexity found in MM makes it impossible to discern an MGUS from a MM clonal cell. Thus, why some of them progress to MM and how the malignant progression occur remains poorly understood. The lapse from MGUS diagnosis to MM diagnosis or associated disorder can vary widely, from 1 to 32 years, with a median of 10.4 years according to a long-term follow-up of MGUS patients performed by the Mayo Clinic [7]. It is widely accepted that the risk of progression is constant regardless of the number of years that a patient has been suffering from MGUS. This feature suggests that once MGUS has been established, a second independent event may be needed. Precisely, one of the main focuses of the MM research field is to study the risk factors and molecular mechanisms behind MGUS progression. Some of the events proposed in the literature include new translocations, increased bone marrow angiogenesis, changes in cytokine expression patterns, or changes in immunity, among others. However, no treatment to prevent this outcome has been considered effective and the reason for a long-lasting MGUS status is unknown. Apart from age, there are other known risk factors for MGUS progression including familial aggregation, male predisposition, race (higher risk in African American and African population), and obesity.

The bone microenvironment is an appropriate site to study changes that could possibly drive controlled clonal plasma cells into a more malignant monoclonal gammopathy. It is well known that osteoclasts increase their activity during MM, releasing growth factors and cytokines that promote myeloma proliferation [8, 9]. Bone marrow stromal cells also have a well-characterized role in MM homing to the bone marrow [10]. These are just some examples of the mechanisms responsible for MM proliferation and expansion. Treatments blocking this crosstalk to stop MM progression are already in the clinic, although with limited success. Thus, the bone marrow remains a dynamic niche that clonal B cells can alter to migrate and proliferate to promote tumor survival. Nonetheless, little is known about how the bone marrow microenvironment affects MGUS pathogenesis.

## MGUS: a Gammopathy of Bone Significance

As described earlier, MGUS has always been considered a benign disorder. Although these patients have an important risk of progression to more malignant gammopathies, MGUS status itself was considered a non-threatening condition. More recently, evidence is accumulating to challenge this. Massive bone marrow infiltration occurring in MM leads to an increase in osteoclast activity and an inhibition of osteoblast activation, resulting in increased bone resorption and causing lytic bone lesions. Roughly 80% of MM patients suffer a fracture during their disease, often being the first sign to gain clinical consideration and generally concurring with the time of diagnosis [11–13]. Interestingly, Melton et al. described a significant increase in overall fracture risk for MGUS individuals mainly due to fractures in axial skeletal sites (predominantly vertebrae), but not in the peripheral skeleton. The etiology behind these results was not clarified. The study was performed using a small cohort of 488 MGUS patients, with limited racial and geographical distributions, so generalization of this data presents some limitations [14]. The Mayo Clinic also performed a study with 605 MGUS patients showing an increased risk in vertebrae, hip, and clavicle fractures but again with no higher risk found for distant long bone fractures [15]. A larger Danish study including 1535 patients also concluded a higher risk of fractures although the risk was lower compared to previous studies, likely reflecting a lower average age of the cohort [16]. Kristinsson et al. studied a much bigger cohort of data from 5326 MGUS patients showing that axial sites were presenting a higher risk of fracture but also distal bones were susceptible [11]. Hematopoietic marrow is mainly produced in axial bones and therefore the authors of these studies suggest that this mechanism might be behind the increased risk of fractures. Discrepancies regarding distant sites risk and the exact level of risk for fractures can be explained by the heterogeneity and the size of each cohort. Little is known about the outcome of the patients presenting fractures in these studies. Whether these patients were misclassified as MGUS or were in incipient MM transformation is not clear. More recently, Thorsteinsdottir et al. published a new study using the Age, Gene/Environment Susceptibility-Reykjavik Study (AGES-RS), a population-based cohort [17]. They propose that the use of a cohort based on a population screening is more representative than those studies performed with patients incidentally diagnosed when reaching the clinic with underlying conditions. Interestingly, they showed by quantitative computerized tomography (QCT) that contrary to previous publications, MGUS patients had no decrease in bone mineral density (BMD) but an increase in bone volume in lumbar spine, femoral neck, trochanter, and total hip. Surprisingly, men had the higher increase in bone volume and also a significant increased risk of fractures suggesting that the mechanism behind MGUS bone alterations is not explained by osteoporosis [17]. While other studies have also found an increase in bone size, this was explained as a compensatory mechanism to overcome the increase in endosteal bone resorption [12••, 18•].

For many years, MGUS diagnosis has been characterized by the fulfillment of certain criteria, one of which was not to present bone lesions. Recently, more evidence is suggesting this is no longer the case, with several studies showing skeletal impairment even before the progression to MM (Table 1). However, some controversy is revealed by these population-based studies. High-resolution peripheral quantitative computed tomography (HRpACT) images were used in a cohort of MGUS patients to show that compared to age, sex, height, and body mass index-matched controls, individuals with MGUS presented significant changes in bone architecture [12••, 18•, 19]. These included decreased cortical and trabecular thickness, lower bone mineral density, and higher cortical porosity. Some mechanism has been proposed for these outcomes. Dickoppf-related protein 1(DKK1) is an antagonist of the Wnt signaling, key for osteoblast differentiation and commitment. Alvin C. Ng et al. showed that levels of DKK1 were higher in MGUS patients compared to control as well as MIP-1α (a potent osteoclast stimulatory factor) suggesting that this could be the reason for a compromised bone formation [12••].

**Table 1:** MGUS patients exhibit changes in bone parameters

Cohort	Findings in MGUS patients	Reference
Olmsted County, Minnesota	Increased overall risk of fractures, attributable to an increase of axial but not limb fractures Slight increase of hip fracture	[14]
North Jutland County, Denmark	Overall risk of fractures	[16]
Swedish population database	Increased risk of fractures: vertebral/pelvis, sternum/costal, arm, or leg	[11]
Mayo Clinic patients	No significant bone density reduction Lower average vBMD and cortical vBMD Lower trabecular and cortical thickness Decreased aBMD at femoral neck and total femur but not at other sites	[12••]
Olmsted County, Minnesota	Decreased cortical vBMD Increased cortical porosity Decreased apparent modulus (bone strength)	[18•]
Reykjavik Study cohort	No differences in spine BMD Increased bone volume in spine and hip No overall risk of fractures (increased just in men population) Increased cortical volume and integral volume of femoral neck area	[17]

Despite current conflicting results, the links between MGUS, bone metabolism alterations, and increased risk of fracture are evident. Nonetheless, a number of limitations surround all these studies. Firstly, the techniques used have considerably different resolutions (dual-energy x-ray absorptiometry (DXA), QCT, HRpQCT). Secondly, the type of cohorts used. The size and heterogeneity of the cohorts are generally quite limited, including patients from geographically restricted areas or similar racial backgrounds. The mean age of these cohorts is also critical. And finally, follow-up information from MGUS patients is normally limited. That is sometimes inevitably intrinsic to MGUS disease, where it is impossible to know for how long patients have been presenting with MGUS criteria, a factor that is probably crucial to understand when bone alterations begin. In other cases, the lack of further follow-up makes it impossible to know how many of them ultimately underwent malignant transformation.

## Does MGUS Subtype Matter?

One of the main diagnostic criteria for MGUS is elevated serum M-protein. Interestingly, the concentration and type of monoclonal protein present in MGUS patients have been found to predict the risk of progression. Patients with IgM or IgA monoclonal protein have increased risk of progression as compared to those presenting serum IgG M-protein. Also, the lower Ig concentration at the moment of diagnosis, the lower the risk of progression [2, 5, 20, 21]. In contrast, no clear association has been found between monoclonal protein concentration or type and skeletal changes. Whereas some studies revealed a lower risk of fractures among IgG MGUS patients compared to other types [14], some others showed higher risk for IgG and IgM [11, 16]. Efforts have also attempted to elucidate a correlation with light chain type, regardless of the heavy chain type. MGUS patients with kappa light chain appear to have increased risk of fractures compared to these with lambda chain [14]. Further investigation needs to be done in this field since there is no evident mechanism for these outcomes and data from MM patients are not supportive of these results.

## Contrasting the MGUS and MM Bone Microenvironment

Progression and clonal plasma cell infiltration in MM has been greatly studied. Osteoblasts, osteoclasts, stromal cells, and immune cells together with other cell types constitute the cellular compartment of the bone marrow, which also includes the vasculature. Together with the cellular compartment, the bone marrow is formed by the extracellular matrix and the soluble compartment. Each of these has been described to play critical roles in myeloma cell migration and proliferation. Even myeloma dormancy and therapy resistance have been proven to occur partially due to changes in the bone microenvironment surrounding myeloma cells [22]. Among all cell types, osteoblast and osteoclasts are key players of the “vicious cycle,” a process that occurs when myeloma cells invade and remodel the bone marrow. It is well established that myeloma cells are able to induce osteoclast activation while inhibiting osteoblast function. Increased bone degradation leads to MM-characteristic lytic bone lesions and to the release of important cytokines and growth factors that myeloma cells use to proliferate and expand. However, much less is known about how these cell types contribute to MGUS pathogenesis. Could osteoblasts and osteoclasts be already affected in MGUS patients? Might it be that initial alterations in osteoblast and/or osteoclast activity, differentiation, or function allow the early stages of B cell clonal expansion occurring in MGUS? Certainly, the aforementioned data about MGUS and skeletal changes supports this notion.

Josselin N and colleagues used specific osteoclast TRAcP staining on sections from ilium biopsies to demonstrate that MGUS patients have a significant increase in osteoclast number compared to healthy individuals [23]. Yet, once again, the nature of the cohort makes difficult to elucidate if that is a cause or a consequence.

RANKL is a member of the tumor necrosis factor family and is the primary molecule responsible for osteoclast differentiation and bone resorption. Because OPG acts as a decoy receptor for RANKL, the balance between RANKL and OPG indicates the overall level of RANK activation within that environment. Both RANKL and RANKL/OPG levels are known to be increased during MM contributing to the “vicious cycle” occurring in MM. Interestingly, RANKL and RANKL/OPG levels are already increased in high-risk MGUS patients suggesting that although no lesions can be detected, osteoclasts might play an important role in MGUS pathogenesis [24, 25].

It has been widely studied how bone marrow cells (and specifically osteoblasts) can play a role in the regulation of hematopoietic stem cells (HSCs) and B-lymphopoiesis [26–28]. Moreover, it is known that HSCs reside in unique niches within the bone marrow controlling their migration, dormancy, and self-renewal [27, 29]. Recently, research has focused on how cancer cells also engraft to these HSC niches promoting migration and dormancy of the cancer cells. Yet again, osteoblasts are considered the main regulators of the bone marrow tumor niche. Several studies have focused on specifically understanding if cells within the bone marrow can have an initial role in hematologic diseases or tumor-induced bone lesions. Kode A. et al. elegantly showed how a mutation triggering β-catenin overexpression in osteoblast could induce acute myeloid leukemia, developing common chromosomal aberrations [30••]. They further identified that FoxO1 interacts with β-catenin in the osteoblast to induce the expression of Notch ligands (Jagged-1) subsequently activating Notch signaling in HSCs [31]. Thus, they demonstrate how mutations in osteoblasts can produce changes in the fate of HSCs and so induce an oncogenic process.

This key role of osteoblasts has not only been seen in hematologic cancers but also recently in tumor invasive ones as prostate cancer. Disseminated prostate cancer cells compete with HSCs for bone niches to establish in the bone marrow and metastasize [32, 33]. Two-photon microscopy has been extremely useful to track cancer cells homing into the bone marrow. Using this technique, Wang et al. demonstrated that prostate cancer cells preferentially migrate to osteoblast-rich areas in early stages of metastasis suggesting these regions as a target for new therapies aiming to prevent bone metastasis [34].

MM has been much less studied from this perspective. There is now evidence revealing how quiescent/dormant myeloma cells also reside in osteoblast niches [22, 35]. However, no specific early mutations from bone marrow cells have been described to promote early stages of MGUS or MM itself. Some studies have compared mesenchymal stem cells (MSCs) isolated from healthy and MM patients showing that MSCs are genetically different after some days in culture, suggesting that MSCs are permanently altered even when myeloma cells are removed from the environment [36].

Altogether, evidence from this and other research fields suggests that osteoblast and other cells within the bone marrow microenvironment could potentially be driving early mutations present in the plasma cells of MGUS patients. Which genetic alterations in BM cells are necessary for plasma cells to start their path to oncogenic cells remain unknown and limitations in MGUS patient cohorts further complicate our understanding of these early events.

## Bone Marrow Adiposity in MGUS and MM

In 2016, the World Health Organization (WHO) considered that there was enough evidence to associate body fatness and MM [37]. Research has also documented that high-fat diet-induced obesity produces a myeloma-like condition in non-permissive C57/B6 mice [38]. However, whether and/or how obesity is related to an increase in MGUS predisposition or to a higher risk of MGUS progression to MM is unclear. To date, just a few studies with patients have assessed the relationship between obesity and MGUS, with conflicting results. Whereas one study shows increased risk of MGUS in patients with body mass index (BMI) higher than 30, other studies show no association between MGUS and obesity even when including obesity measures other than BMI. Similarly to MGUS and risk of fracture studies, discrepancies in the association of MGUS risk and obesity can rely on the nature of the cohorts. However, obesity is known to be a risk factor for progression [39–41].

Together with several cytokines and growth factors, circulating adipokines have also been examined in myeloma patients. Adiponectin has been shown to have anti-tumor effects and it is decreased in patients with cancer from different origins, including MM. Even when compared to MGUS, MM patients have a significant decrease in circulating adiponectin [42–44]. Interestingly, serum adiponectin levels decrease in obesity, thought to be due to dysfunctional adipocytes [45, 46]. Whether these adipocytes are already altered in MGUS patients allowing the initial invasion of malignant plasma cells within the bone marrow is unknown.

Bone marrow adipocytes (BMAs) are cells within the bone microenvironment that have recently become an important field of study in myeloma research due to their significant metabolic effects. BMAs have a very active role within the bone marrow by releasing several cytokines and adipokines (such as IL-6 and adiponectin) and potentially inducing changes in adjacent cell types, including invasive clonal plasma cells [46]. BMAs are increased in response to several processes such aging or anorexia nervosa and either calorie restriction or high-fat diet can increase bone marrow adiposity in mice [47–50]. The fact that bone marrow adiposity can be at least partially modulated gives hope for new therapies and/or dietary interventions. Interestingly, Trotter TN et al. showed how myeloma cells that were previously in contact with adipocyte-lineage cells presented higher tumor progression when later injected in the myeloma-permissive mice C57/KaLwRij mice [51]. However, these results were achieved using non-bone marrow-derived adipocytes and further studies need to be performed in order to completely clarify the specific role of BMAs within the MM bone microenvironment [46, 52]. As the BMAs and MM relationship is just beginning to be elucidated, we are even further away from understanding bone marrow adiposity role in MGUS. Nonetheless, sufficient evidence is available to consider whether potentially dysfunctional BMAs could drive changes creating a more hostile microenvironment for initial malignant cells to engraft.

## Conclusions

MM evolves from the premalignant disorder termed MGUS. It has recently become clear that MGUS is no longer the symptomless condition that it was considered. As discussed in this review, bone density alterations and higher risk of fractures are some of the consequences of this condition. Additionally, it has been reported that MGUS cohorts have a lower life expectancy compared with sex- and age-matched population controls, which is not completely explained by the risk of malignant transformation [1, 21, 53]. Although MGUS is considered non-malignant, this does not mean that it does not have any clinical significance. Bone is not the only tissue that seems to be affected by MGUS. In fact, new terminology as monoclonal gammopathy of renal significance (MGRS) has begun to be used. MGRS define patients with renal disorders attributed to plasma cell proliferation although still being closer to MGUS classification than to MM in terms of cell proliferation rate and invasion [54].

MGUS patients are often accidentally diagnosed due to a concomitant clinical disorder. With regard to clinical studies, that leads to heterogeneous cohorts of patients that vary in years since early clonal malignancy. In addition to the limited research and clinical data about MGUS available, the molecular mechanisms underlying MGUS are not fully understood, in part due to the limitations of in in vivo and in vitro models. As detailed in this review, MGUS and MM are highly dependent on their microenvironment for establishment and progression. The level of cellularity and complexity of the bone marrow microenvironment is not achievable in vitro which leaves us with a poor understanding of what realistically happens physiologically. There are a range of preclinical models mimicking MM progression [55]. However, the lack of an ideal mouse model to reproduce monoclonal gammopathies such as MGUS makes it difficult to gain knowledge about the early stages of this condition. Recently, a new genetically humanized mice model has been developed, reproducing pre-neoplasic conditions [56]. The future development of models like this will be extremely useful to advance the study of MGUS pathogenesis and to better understand the initial events of malignant transformation that ultimately will help us to develop drugs for targeting the microenvironment and/or its interaction with clonal plasma cells.
